# Comment on Zwierz et al. The Long-Term Effects of 12-Week Intranasal Steroid Therapy on Adenoid Size, Its Mucus Coverage and Otitis Media with Effusion: A Cohort Study in Preschool Children. *J. Clin. Med.* 2022, *11*, 507

**DOI:** 10.3390/jcm11071983

**Published:** 2022-04-02

**Authors:** David Kalfert

**Affiliations:** Department of Otorhinolaryngology and Head and Neck Surgery, First Faculty of Medicine, University Hospital Motol, Charles University, 15006 Prague, Czech Republic; david.kalfert@fnmotol.cz; Tel.: +420-22443-4326

I read with great interest the article entitled “The Long-Term Effects of 12-Week Intranasal Steroid Therapy on Adenoid Size, Its Mucus Coverage and Otitis Media with Effusion: A Cohort Study in Preschool Children” by Zwierz et al. [1]. This is an excellent study. 

In this study, the authors used examination adenoid hypertrophy according to Boleslavska et al. [2,3]. This classification was not completely used. It considers the anatomical relationships between adenoid tissue, choanal obstruction, and torus tubarius [2,3]. 

In this regard and according to choanal obstruction, adenoids are differentiated into three grades:

- Grade I: adenoid tissue that fills less than one third of the vertical portion of the choanae;
- Grade II: filling of the adenoid tissue from one third to two thirds of the choanae;
- Grade III: adenoid tissue that fills more than two thirds of the choanae.

The condition of the nasopharyngeal orifice of the Eustachian tube was also differentiated into three grades related to the condition of adenoid tissue:

- Grade A: adenoid tissue not in contact with the torus tubarius;
- Grade B: adenoid tissue in contact with the torus tubarius without complete covering;
- Grade C: adenoid tissue that completely covers the torus tubarius.

I do not understand how they performed the classification of the size of the adenoids for the patients in the study. Why did the authors not classify the conditions of adenoid tissue into the nasopharyngeal orifice of the Eustachian tube? In otitis media with effusion, the relation between adenoids and torus tubarius is more important than the volume of the adenoids [3].
